# HypoxyStat, a small-molecule form of hypoxia therapy that increases oxygen-hemoglobin affinity

**DOI:** 10.1016/j.cell.2025.01.029

**Published:** 2025-02-17

**Authors:** Skyler Y. Blume, Ankur Garg, Yolanda Martí-Mateos, Ayush D. Midha, Brandon T.L. Chew, Baiwei Lin, Cecile Yu, Ryan Dick, Patrick S. Lee, Eva Situ, Richa Sarwaikar, Eric Green, Vyas Ramanan, Gijsbert Grotenbreg, Maarten Hoek, Christopher Sinz, Isha H. Jain

**Affiliations:** 1 Gladstone Institutes, San Francisco, CA 94158, USA; 2 Department of Biochemistry and Biophysics, University of California, San Francisco, San Francisco, CA 94158, USA; 3 Maze Therapeutics, 171 Oyster Point Blvd STE 300, South San Francisco, CA 94080, USA; 4 Arc Institute, 3181 Porter Dr, Palo Alto, CA 94304, USA; 5 Lead contact

## Abstract

We have previously demonstrated that chronic inhaled hypoxia is remarkably therapeutic in the premier animal model of mitochondrial Leigh syndrome, the *Ndufs4* knockout (KO) mouse. Subsequent work has extended this finding to additional mitochondrial diseases and more common conditions. However, challenges inherent to gas-based therapies have hindered the rapid translation of our findings to the clinic. Here, we tested a small molecule (hereafter termed HypoxyStat) that increases the binding affinity of hemoglobin for oxygen, thereby decreasing oxygen offloading to tissues. Daily oral dosing of HypoxyStat caused systemic hypoxia in mice breathing normoxic (21% O_2_) air. When administered prior to disease onset, this treatment dramatically extended the lifespan of *Ndufs4* KO mice and rescued additional aspects of disease, including behavior, body weight, neuropathology, and body temperature. HypoxyStat was also able to reverse disease at a very late stage, thereby serving as a clinically tractable form of hypoxia therapy.

## In brief

A small molecule that increases oxygen-hemoglobin affinity was shown to prevent and reverse pathology in a mouse model of mitochondrial disease, thereby enabling a practical form of hypoxia therapy.

## Graphical abstract

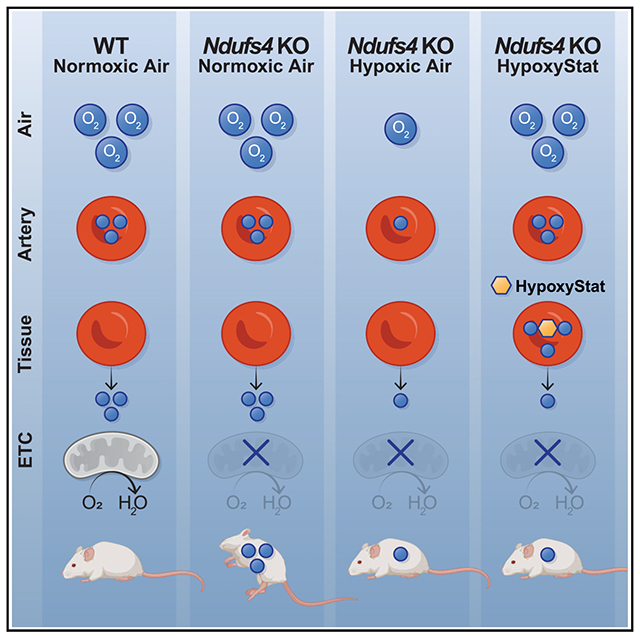

## INTRODUCTION

Oxygen serves as a substrate for over 200 biochemical reactions, making it essential for human health.^1^ However, excess oxygen is also toxic.^2^ We recently demonstrated that mitochondrial diseases reduce whole-body oxygen consumption, leading to an imbalance between oxygen supply and demand.^3–5^ This imbalance results in tissue hyperoxia, as observed in the leading mouse model of mitochondrial disease, the *Ndufs4* knockout (KO) mouse. This model lacks an essential complex 1 subunit of the electron transport chain (ETC) and reproduces the pathology of Leigh syndrome, the most common pediatric mitochondrial disease. Similar findings of hyperoxia are evident in mitochondrial disease patients that exhibit elevated venous oxygen levels due to impaired tissue oxygen extraction.^6^ Notably, we have shown that chronic exposure to inhaled hypoxia (equivalent to an altitude of 4,500 m) normalizes this tissue hyperoxia and dramatically extends the lifespan of *Ndufs4* KO mice.^3,5^ Remarkably, this intervention can even reverse neurological lesions at the late stages of disease.^4^

Recent studies have further highlighted the therapeutic potential of hypoxia in mitochondrial disorders. For example, hypoxia has been shown to mitigate motor defects in a Friedreich’s ataxia mouse model.^7^ Additionally, our genome-wide CRISPR screen comparing hypoxia with normoxia identified over 75 additional monogenic disorders that could potentially benefit from hypoxia therapy.^8^ Beyond inborn errors of metabolism, we recently demonstrated that chronic hypoxia alleviates many aspects of metabolic syndrome.^9^ These findings align with epidemiological data showing a reduced incidence of cardiovascular disease, obesity, and diabetes in populations living at high altitudes.^10–18^ Taken together, these results suggest that hypoxia may represent a therapeutic approach for a broad range of diseases, from rare genetic disorders to more common conditions.

Building on these discoveries, phase 1 clinical trials were recently completed to assess the feasibility of inhaled hypoxia therapy.^19^ Patients were gradually acclimated to hypoxic conditions until they reached an arterial oxygen saturation (SaO_2_) of ~85%. This exposure was well-tolerated, consistent with the fact that humans have resided at altitude for centuries.^20^ Nevertheless, maintaining patients in a state of chronic hypoxia presents significant logistical challenges, underscoring the need to develop more practical approaches to hypoxia-based therapies.

Our previous research indicated that patients with mitochondrial disease would likely require continuous hypoxia throughout most of the day, making intermittent hypoxia or approaches such as “sleeping in hypoxia” insufficient.^4^ Although relocating to high-altitude regions could theoretically provide the required level of hypoxia, this would necessitate living in remote and geographically restricted areas. Alternatively, living spaces could be made hypoxic using nitrogen generators, which separate normoxic room air (21% O_2_) into hypoxic and hyperoxic gas streams.^21^ Such systems are commercially available for athletes training in simulated high-altitude conditions. However, this approach would greatly impact quality of life, as it requires confinement to enclosed spaces such as a room or bassinet. Portable devices delivering hypoxic gas mixtures through nasal cannulas or masks, similar to portable oxygen tanks, might offer greater mobility. However, these devices carry significant risks, as equipment malfunctions could result in dangerously low-oxygen levels and potential fatalities. Consequently, we sought to develop a more practical and safer intervention to achieve sustained tissue hypoxia.

To explore alternative strategies for inducing tissue hypoxia, we revisited the textbook equation for oxygen delivery to tissues.^22^ Inhaled hypoxia reduces hemoglobin (Hb) oxygen saturation in arterial blood, thereby decreasing oxygen delivery to tissues and causing hypoxia. However, several other variables in the oxygen delivery equation can be manipulated to achieve similar effects. For example, we previously demonstrated that reducing tissue oxygen delivery by lowering total Hb content through therapeutic phlebotomy significantly extended the lifespan of *Ndufs4* KO mice.^5^ Similarly, administering low-dose carbon monoxide, which competes with oxygen for Hb binding, also provided protection.^5^ These findings suggested that interventions capable of inducing tissue hypoxia—beyond inhaled hypoxia—could have therapeutic benefits.

**Figure 1.**
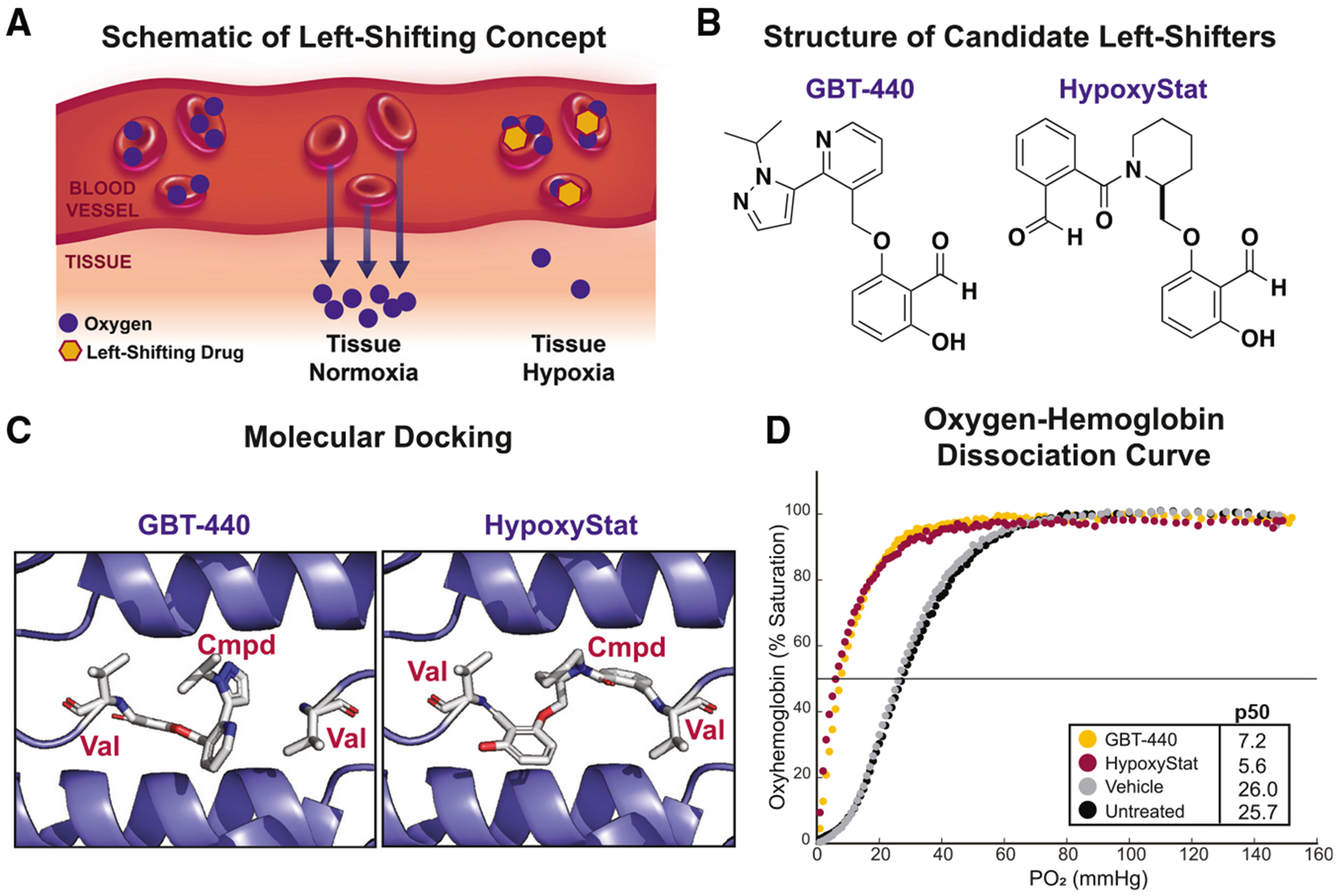
HypoxyStat binds hemoglobin and increases oxygen-binding affinity (A) Schematic of left-shifting concept. Increased Hb-O_2_ binding affinity decreases the release or “offloading” of oxygen to tissues, resulting in relative tissue hypoxia. (B) Structures of GBT-440 and HypoxyStat. (C) Known structure of GBT-440 bound to Hb (adapted from Metcalf et al.^25^) and modeled structure of HypoxyStat bound to Hb. (D) Oxygen-hemoglobin dissociation curve of red blood cells incubated with vehicle, 10 mM GBT-440, or 10 mM HypoxyStat for 1 h. Traces are representative of triplicate experiments. See also Figure S1.

Building on this, we focused on the oxygen-binding properties of Hb. The oxygen-Hb dissociation curve, which describes the relationship between oxygen saturation and local oxygen tension, has a sigmoidal shape. This shape enables efficient oxygen loading onto Hb in high-oxygen environments (e.g., lung alveoli) and oxygen offloading in low-oxygen environments (e.g., internal organs such as the brain) (Figure 1A). We hypothesized that by increasing Hb’s binding affinity for oxygen (a leftward shift in the dissociation curve), and thereby reducing oxygen offloading to tissues, we could induce tissue hypoxia^23,24^ (Figure 1D). This strategy could serve as a small-molecule approach to hypoxia therapy.

## RESULTS

### Concept of left-shifting small molecules as a form of hypoxia therapy

Several physiological variables can result in a left-shift of the oxygen-Hb dissociation curve (ODC), including increased pH, decreased temperature, reduced 2,3-diphosphoglycerate (2,3-DPG) levels, carbon monoxide, and fetal Hb.^23,24^ Leveraging this principle, small molecules can be designed to increase Hb-oxygen-binding affinity. Significant pharmacological efforts have been directed toward developing left-shifting molecules for an unrelated condition, sickle cell anemia. In this condition, red blood cells carrying oxygenated Hb are less likely to sickle than those with deoxygenated Hb. By increasing the likelihood of Hb remaining in the oxygenated state, left-shifting small molecules reduce the proportion of sickled cells and alleviate the associated clinical complications.^25^

**Figure 2.**
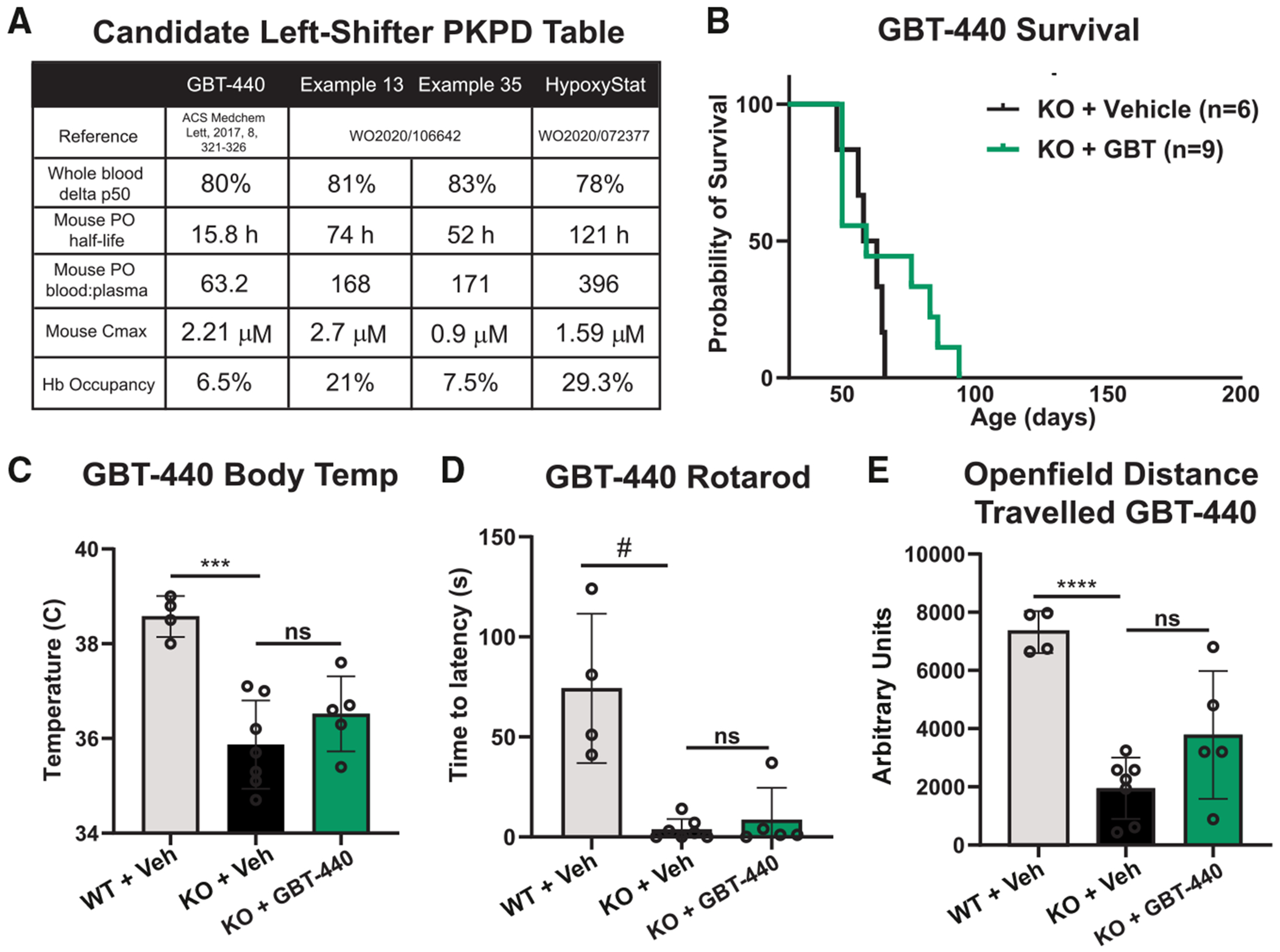
GBT-440 is unable to rescue *Ndufs4* KO survival, body temperature, or behavior (A) PK/PD data for GBT-440 vs. other candidate small-molecule left-shifters, including HypoxyStat. (B) Kaplan-Meier survival curve of mice treated daily with vehicle vs. GBT-440 at the maximum-tolerated dose (chronic 400 mg/kg after 1 week of adaptation at 200 mg/kg). (C) Body temperature of KO mice at post-natal day ~P55 treated with vehicle vs. GBT-440 does not show a significant rescue. (D and E) (D) Time on accelerating rotarod or (E) spontaneous movement during open-field test similarly do not show a significant rescue of *Ndufs4* KO mice by maximum-tolerated dose of GBT-440. #*p* < 0.1, *p < 0.05, **p < 0.01, ***p < 0.001, ****p < 0.0001, ANOVA.

To identify suitable candidates for our study, we reviewed the literature for left-shifting small molecules that exhibited maximal efficacy while retaining favorable drug-like properties. One of the most extensively studied compounds for sickle cell anemia is GBT-440 (Voxelotor) (Figure 1B).^25^ The extent of left-shifting can be assessed by determining the p50 or the partial pressure of oxygen at which half of the total Hb pool is bound to oxygen. We incubated human red blood cells with GBT-440 and observed a ~75% decrease in p50 (from ~28 to ~7 mmHg), consistent with previously reported values (Figure 1D). Experiments were performed using a HemOx analyzer that monitors oxygen saturation via Hb absorbance across a range of dissolved oxygen tensions. Upon further review of the sickle cell anemia literature, we found another compound, hereafter termed HypoxyStat (Figure 1B), which appeared to have a similar p50 as GBT-440.^26^ We confirmed that HypoxyStat induced an ~82% decrease in p50 (Figure 1D). Notably, this compound was reported to have a pharmacokinetic profile more suitable for our needs than GBT-440, including a longer half-life and greater blood/plasma ratio, which is an indirect measure of target engagement (Figure 2A).^25,26^ Upon oral administration of HypoxyStat (10 mg/kg oral gavage) to C57BL/6 mice, we observed an effective half-life of 121 h and a blood/plasma ratio of 396 at 3 days post dosing (Figure 2A). These pharmacokinetic and pharmacodynamic (PK/PD) properties in mice suggested that HypoxyStat was more likely than GBT-440 to induce chronic hypoxia in our mouse model.

Previous studies have reported the structure of GBT-440 bound to Hb.^25^ We used the GBT-440 structure to computationally model HypoxyStat in the binding pocket and then applied restrained minimization (Figure 1C). This modeling suggests that HypoxyStat has the potential to form Schiff bases with the N-terminal valines of both the α_1_- and α_2_-subunits of Hb, as opposed to the single binding site of GBT-440, providing a potential rationale for the increased half-life and enhanced partitioning into red blood cells.

### Established left-shifting compound GBT-440 is unable to rescue *Ndufs4* KO mice when administered alone

We first tested our prediction that GBT-440 would not be sufficient to rescue *Ndufs4* KO mice based on its half-life and red blood cell distribution. Indeed, daily maximal dosing of GBT-440 starting post-natal day 30 (P30) (before disease onset) did not result in a significant lifespan extension compared with vehicle (Figure 2B). It also failed to rescue body temperature (Figure 2C), time on an accelerating rotating rod (Figure 2D), or spontaneous movement (Figure 2E). Based on these results, we proceeded with characterizing our lead candidate, HypoxyStat.

### HypoxyStat PK/PD and safety data

**Figure 3.**
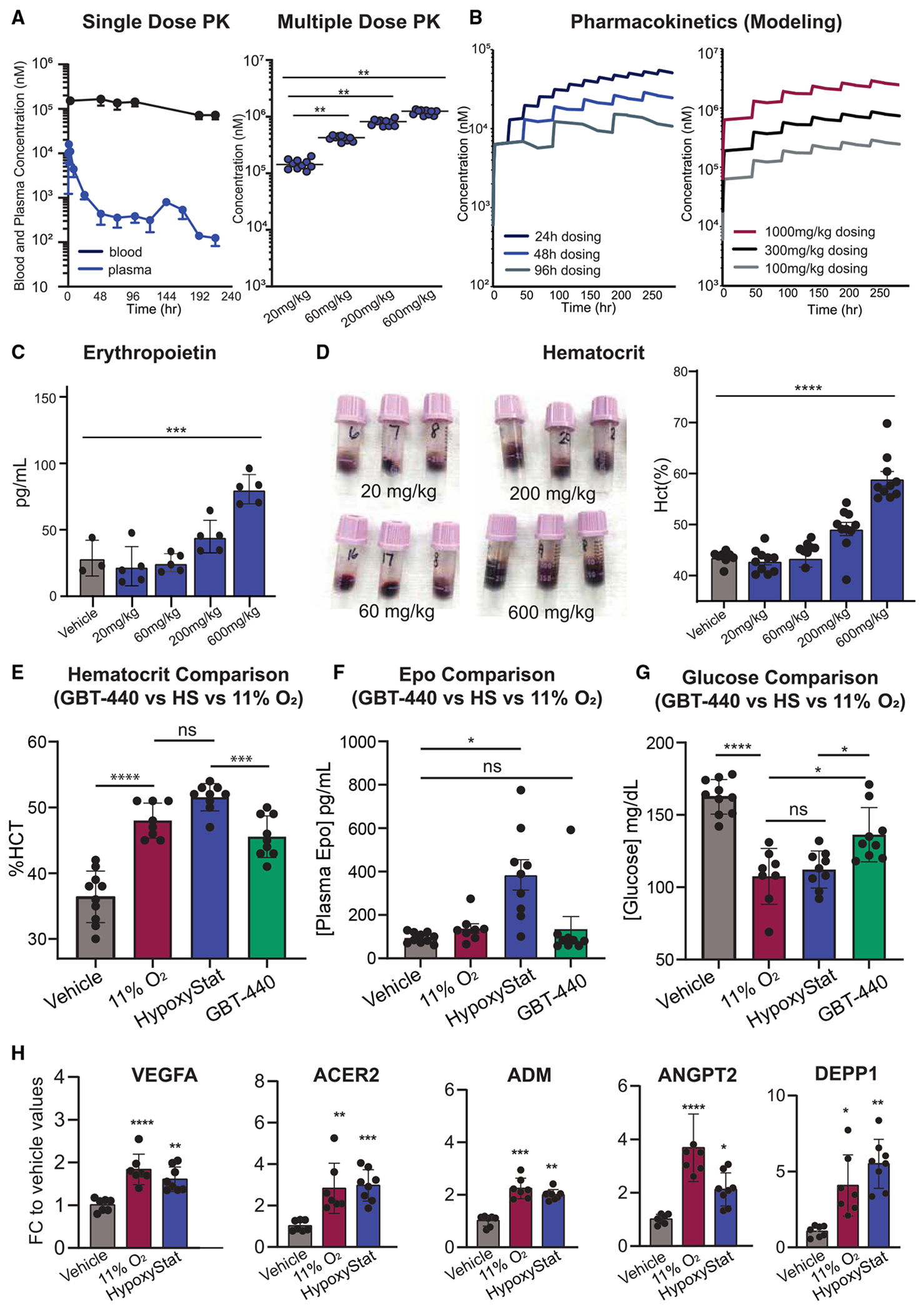
HypoxyStat administration results in tissue hypoxia comparable with inhaled hypoxia (A) Left panel: blood and plasma pharmacokinetic profile of HypoxyStat following single oral administration in C57BL/6 mice (10 mg/kg in 0.5% HPMC). Right panel: blood levels of HypoxyStat following 12-day administration in C57BL/6 mice (20, 60, 200, and 600 mg/kg, 3 times per week in 0.5% HPMC). (B) Pharmacokinetic modeling of HypoxyStat at various schedules and doses. (C and D) (C) Plasma Epo concentration (*n* = 3+) and (D) hematocrit (*n* = 5+) following administration of HypoxyStat at 600 mg/kg, 3 times per week, in 0.5% HPMC for 12 days. (E–H) (E) Hematocrit, (F) plasma Epo, (G) blood glucose, and (H) five hypoxia-induced transcripts in the brain measured post daily administration of vehicle or HypoxyStat (400 mg/kg) or initiation of continuous 11% O_2_ breathing. This was done after 2 days (plasma Epo, HIF targets) or after 2 weeks (hematocrit, blood glucose). *p* values relative to vehicle. HypoxyStat and inhaled hypoxia have similar effect sizes, whereas GBT-440 is less effective. *p < 0.05, **p < 0.01, ***p < 0.001, ****p < 0.0001, ANOVA. See also Figures S2 and S3.

We conducted a PK/PD study to determine the optimal dosing strategy for HypoxyStat in a chronic setting. First we administered a single 10 mg/kg oral dose of HypoxyStat to adult C57BL/6 mice and monitored its concentration in blood and plasma over a 10-day period (Figure 3A, left). Next, we administered HypoxyStat at doses of 20, 60, 200, and 600 mg/kg via oral gavage three times per week for 12 days. This regimen resulted in a dose-dependent increase in HypoxyStat concentration within red blood cells (Figure 3A, right). Based on blood levels of HypoxyStat and Hb measured on day 12, we estimated Hb occupancy to be 29.3% at 200 mg/kg and 41.4% at 600 mg/kg (Figure S1).^27^

To further refine the dosing strategy, we used single-dose PK data to model a range of dosing frequencies and amounts. These simulations were performed using nonparametric superposition in Phoenix 64 (Certara) (Figure 3B). We then assessed the dose-dependent effects of HypoxyStat on two key markers of the hypoxia response: erythropoietin (Epo) levels and red blood cell count (hematocrit). Inhaled hypoxia is known to increase circulating Epo through activation of hypoxia-inducible transcription factors (HIFs) in the kidney. Elevated Epo levels, in turn, stimulate red blood cell production, thereby raising hematocrit. Consistent with this mechanism, we observed substantial increases in both Epo and hematocrit as HypoxyStat doses were increased (Figures 3C and 3D). Based on these findings, we selected a daily dosing regimen of 400 mg/kg for subsequent studies as the most effective and practical dose.

We performed additional safety studies at this optimized dose and demonstrated that classical safety parameters (body weight, organ weights, complete blood count, and clinical chemistries) were not adversely affected by our dosing regimen (Figure S2). Additionally, we used the Reaction Bio InVEST panel to test 25+ potential off-targets that have been identified by a consortium of pharmaceutical companies as high-yield candidates. All tests were negative, suggesting that HypoxyStat does not significantly inhibit or bind such proteins in an off-target manner (Figure S3).

### HypoxyStat results in tissue hypoxia that is comparable to therapeutic inhaled hypoxia

After determining the optimal HypoxyStat dosing strategy, we then performed a head-to-head comparison with inhaled hypoxia (11% O_2_, which demonstrated a therapeutic effect in our past work). This was done by measuring circulating Epo levels (Figure 3F) and 5 different hypoxia-induced transcripts in hindbrain homogenate after 48 h of treatment (Figure 3H). All these biomarkers of the hypoxia transcriptional response were induced to an equivalent or greater degree by HypoxyStat compared with inhaled hypoxia. We also measured the increase in hematocrit (Figure 3E) and decrease in blood glucose (Figure 3G) at ~12 days, both of which are well-established biomarkers of chronic hypoxia.^9^ Both metrics demonstrated that inhaled hypoxia and HypoxyStat have similar effects on classical hypoxia biomarkers. By contrast, GBT-440 elicited a significantly blunted response.

### HypoxyStat results in a 3–4× lifespan extension of *Ndufs4* KO mice

**Figure 4.**
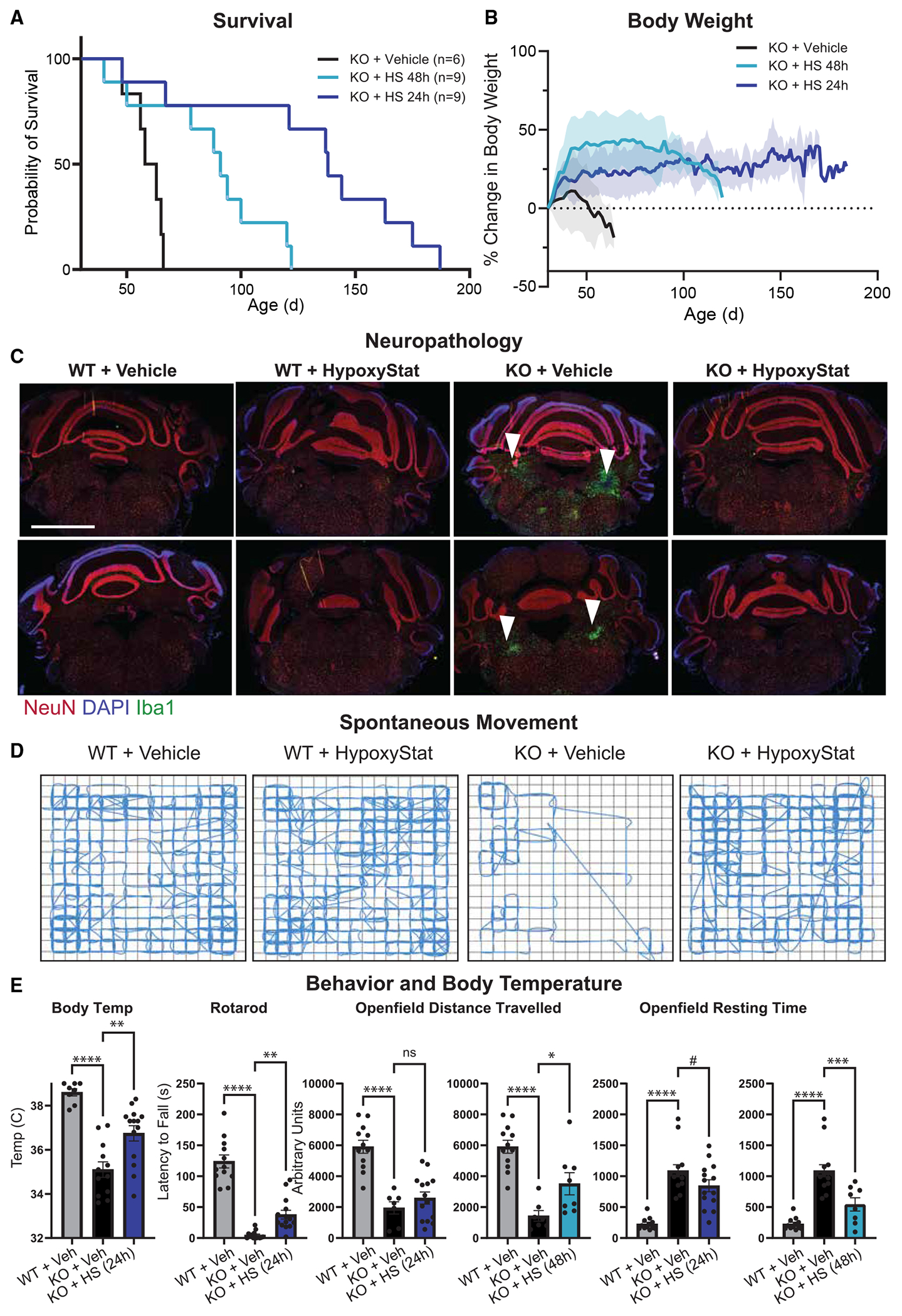
HypoxyStat improves survival and outcomes of *Ndufs4* KO model of Leigh syndrome (A) Kaplan-Meier survival curve of KO mice given vehicle vs. HypoxyStat (every 48 h or every 24 h, light blue and dark blue, respectively). The optimized dosing results in a 3–4× lifespan extension of KO mice. *p < 0.001 by Mantel-Cox test for 24 h dosing relative to vehicle and *p* < 0.01 for 48 h dosing. *n* = 6 (vehicle), *n* = 9 (48 h HypoxyStat), and *n* = 9 (24 h HypoxyStat). (B) Body weight (mean ± SD) of same cohort of mice as (A). (C) Representative neuropathology images of Iba1 staining in WT and KO mice +/− HypoxyStat or vehicle starting at P30. (D) Representative spontaneous movement traces of P50 mice from 48 h dosing (24 h dosing data in Figure S5). (E) Quantification of body temperature, time on accelerating rotating rotarod, and open-field movement in the same experimental groups. #*p* < 0.1,*p < 0.05, **p < 0.01, ***p < 0.001, ****p < 0.0001, ANOVA. See also Figures S4, S5, and S6.

After optimizing dosing strategies, we evaluated the efficacy of HypoxyStat in the *Ndufs4* KO mouse model of Leigh syndrome. Mice were treated either every other day (600 mg/kg) or daily (400 mg/kg) via oral gavage, starting at P30, prior to overt disease onset. For daily dosing, a 1-week acclimation dose of 200 mg/kg was used. Both dosing regimens significantly extended the lifespan of KO mice, with every-other-day dosing achieving a 1.5–2× increase and daily dosing achieving a 3–4× increase (Figure 4A). Importantly, all control mice (wild-type [WT] or heterozygous) survived chronic drug administration, indicating that these dosing regimens are well tolerated. Drug-treated KO mice also exhibited increased body weight, comparable with the effects of inhaled hypoxia (Figure 4B).

Next, we assessed whether HypoxyStat could rescue neuropathology and behavior. Leigh syndrome is characterized by bilateral symmetric lesions in the vestibular nuclei, a hallmark feature seen in both mouse models and human patients. We previously demonstrated that inhaled hypoxia could prevent and reverse these lesions. Similarly, KO mice treated with HypoxyStat from P30 onward showed a significant reduction in lesion severity at P50, as indicated by Iba1 staining for microglial activation (Figures 4C and S4). The percentage of Iba1+ cells was significantly lower in HypoxyStat-treated mice compared with vehicle-treated controls (*p* < 0.05, Student’s t test). By contrast, vehicle-treated KO mice exhibited the expected neuropathology at P50.

HypoxyStat treatment also restored body temperature to normal levels (Figure 4E). Behavioral improvements were assessed using an accelerating rotarod test and an open-field test to measure spontaneous movement (Figures 4D and 4E). Vehicle-treated KO mice showed impaired movement, reflecting muscle weakness, atrophy, and loss of coordination. By contrast, HypoxyStat-treated KO mice displayed significantly improved motor coordination and spontaneous activity (Figures 4D, 4E, and S5; Video S1). There were no observed effects on skeletal muscle morphology (Figure S6). These results suggest that HypoxyStat not only extends lifespan but also rescues key neuropathological and behavioral deficits in the premier mouse model of Leigh syndrome, further underscoring its therapeutic potential.

### HypoxyStat can reverse disease at a very late stage of presentation

**Figure 5.**
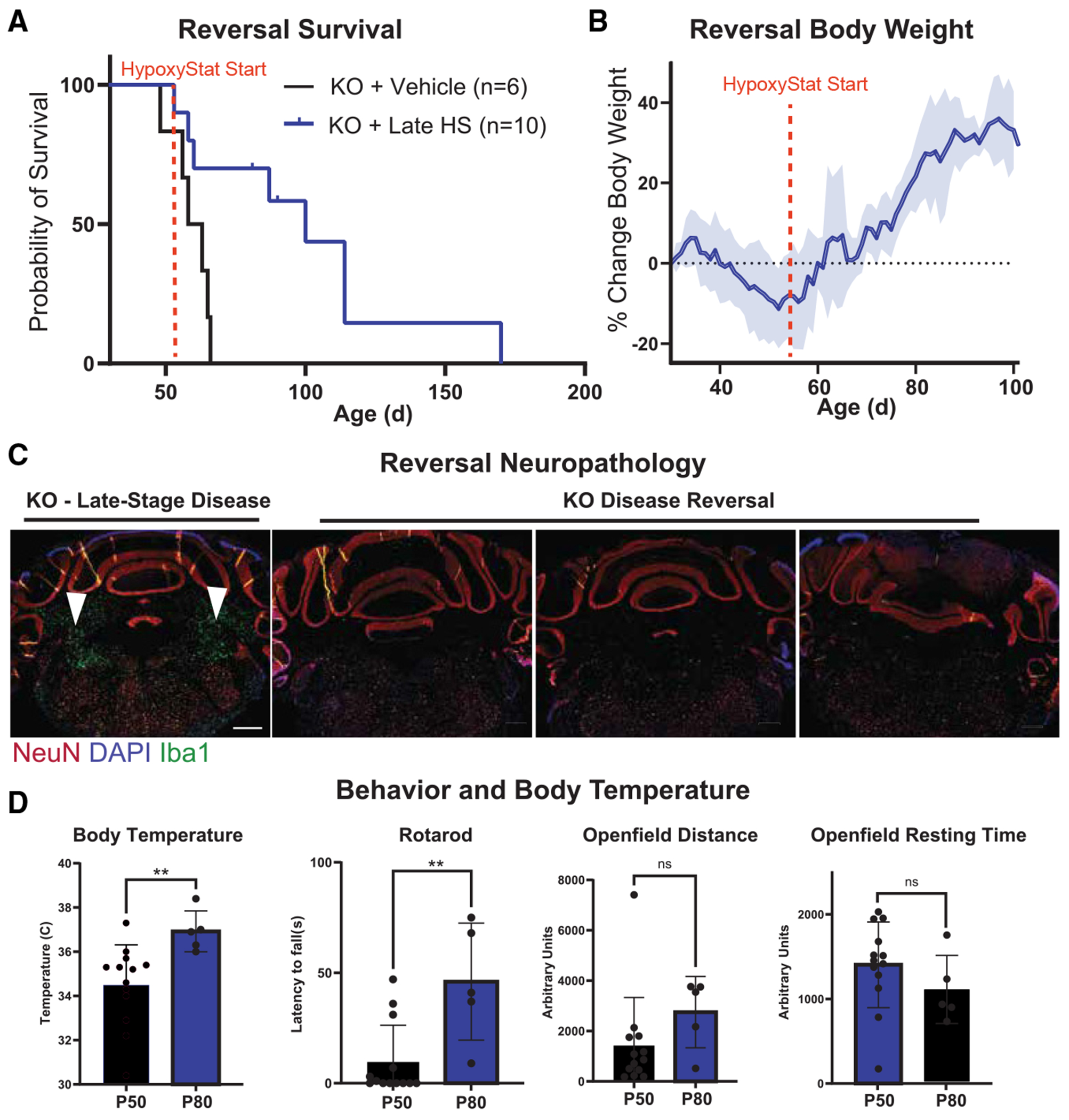
HypoxyStat reverses disease in *Ndufs4* KO mice (A) Kaplan-Meier Survival curve of KO mice treated at a very late stage of disease (once a subset of KO mice have already started dying). *p* < 0.05 by Mantel-Cox test. (B) Body weight (mean ± SD) of KO mice before and after daily treatment with HypoxyStat. (C and D) (C) Neuropathology; (D) body temperature, rotarod, and open-field data for KO mice before (P50) or after initiation of HypoxyStat at late stages of disease (P80). **p < 0.01, unpaired t test.

Mitochondrial disease is often diagnosed after patients experience their first metabolic crisis, highlighting the need for therapeutic strategies that remain effective even at advanced stages of disease. To address this, we tested HypoxyStat in mice at a very late stage of disease (~P50), when major symptoms—extensive neurological lesions, impaired behavior, and low body temperature —are fully apparent. At this time point, some vehicle-treated mice had already begun to die, underscoring the severity of the disease at this stage. Remarkably, initiating daily HypoxyStat treatment at P50 significantly extended the lifespan of these late-stage *Ndufs4* KO mice (Figure 5A). HypoxyStat also reversed multiple other disease presentations, including body weight loss (Figure 5B), impaired behavior (Figure 5D), and neurological lesions (Figure 5C, *p* < 0.05, Student’s t test). These findings demonstrate that HypoxyStat retains robust therapeutic efficacy even when administered after significant disease progression. This ability to reverse advanced pathology positions HypoxyStat and related compounds as promising therapeutic candidates for mitochondrial diseases, where early diagnosis and intervention are often challenging.

## DISCUSSION

This study establishes a proof-of-concept for “HypoxyStat,” a small-molecule form of hypoxia therapy. Our previous work demonstrated the therapeutic potential of inhaled hypoxia for mitochondrial diseases but highlighted significant challenges associated with gas-based therapies, such as logistical and safety concerns, which have hindered clinical translation. The development of HypoxyStat addresses this unmet need, transforming a compelling experimental phenomenon into a practical and translatable therapeutic strategy.

The current small molecule we tested causes a dramatic left-shift of oxygen-Hb binding affinity and results in a 3- to 4-fold lifespan extension in the *Ndufs4* KO mouse model. This is comparable with the effects observed by inhaled hypoxia.^3^ Future work will include screening for next-generation left-shifters with even greater Hb-oxygen-binding affinity and better PK/PD properties (e.g., longer half-life, greater blood-to-plasma exposure, etc.).

One known side effect of hypoxia is increased hematocrit and increased vascular density. These adaptations are triggered by the HIF transcriptional program and serve to increase oxygen delivery during chronic hypoxia, which might partially normalize tissue oxygen levels. To overcome this effect, we can inhibit the HIF2-mediated increase in hematocrit. There are several HIF-2α inhibitors in clinical development and one approved for indications such as renal cell carcinoma (Belzutifan, Merck).^28^ Such inhibitors can block the compensatory effects that partially increase tissue oxygenation in states of chronic hypoxia. Future preclinical work will focus on combining HIF-2α inhibitors with HypoxyStat to further boost efficacy, as recently proposed.^29^

In the case of the *Ndufs4* KO mouse, we found that ~14% inhaled oxygen was the threshold below which a dramatic lifespan extension was observed.^4^ However, the exact degree of hypoxia required may differ between humans and mice. Additionally, the half-life of Hb is longer in humans than mice, thereby requiring lower and less-frequent dosing of HypoxyStat.^30^ Moreover, the concept of hypoxia therapy serves to combat the excess tissue oxygen that builds up due to impaired ETC activity. Thus, diseases stemming from milder forms of ETC deficiency will likely cause milder levels of hyperoxia and require lower doses of HypoxyStat.

It will also be important to consider the safety profile of a left-shifting small molecule. With a gas-based therapy, it is possible to cause extreme hypoxia due to equipment malfunction and incorrect gas mixing. With the current class of left-shifting small molecules, we expect that the maximal effect size will occur when every molecule of Hb is covalently modified with HypoxyStat, thereby creating a limit to the extent of tissue hypoxia that is possible. This is dictated by the left-shifting property and serves as a safety check on the therapeutic approach. However, the covalent nature of the drug-binding also means that this effect cannot be acutely reversed. To counter the effects of HypoxyStat, patients would need blood transfusions with unmodified Hb molecules.

More generally, chronic hypoxia is known to cause pulmonary hypertension, which can lead to right heart failure.^1^ It will be important to assess the long-term safety of HypoxyStat in this context. Just as individuals are advised to ascend slowly to high altitude to allow for gradual adaptations, we envision that gradual dose escalation will be required for left-shifting small molecules. In the future, we plan to perform more extensive safety and toxicology studies in rodent and primate models, using second-generation HypoxyStat molecules.

Although this study focuses on mitochondrial Leigh syndrome, the principles underlying HypoxyStat therapy could extend to other mitochondrial disorders and more common conditions that benefit from hypoxia therapy. Testing HypoxyStat in additional disease models will be critical to broadening its therapeutic scope. This work represents an important next step toward a practical and scalable approach to hypoxia therapy, advancing a paradoxical concept into the realm of clinical translation.

### Limitations of the study

This study focuses on the *Ndufs4* KO mouse model of Leigh syndrome. Future work will be needed to test the generalizability of these findings to additional disease models. Moreover, our current safety studies are primarily in mice or *in vitro*. Future work is needed in primate models to establish the safety and efficacy of HypoxyStat. Although this is our first-generation compound, future drug screens will be required to fully optimize HypoxyStat before it enters human studies.

## RESOURCE AVAILABILITY

### Lead contact

Additional information or requests for resources will be fulfilled by the lead contact, Isha Jain (Isha.Jain@gladstone.ucsf.edu).

### Materials availability

No new materials were created for this study. HypoxyStat was synthesized by WuXi AppTec.

### Data and code availability

No new code was generated for this study. No large datasets were generated for this study.

## STAR★METHODS

### EXPERIMENTAL MODEL AND STUDY PARTICIPANT DETAILS

#### Animal Care

We continuously bred Ndufs4 Het x Het mice to generate KO and controls for our experiments. Ndufs4+/− and WT mice were used as controls as they are identical in all previously reported assays. Ndufs4 mice were obtained directly from Jackson labs (#027058). Pups were weaned and genotyped at ~21 d after birth. The age of mice at the time of experiments is described throughout the text. Male and female mice were used throughout studies. All cages were provided with food and water, with hydrated gel provided to newly weaned cages. Body weights were recorded at every dosing. Mice were euthanized according to guidelines at 20% drop in peak body weight. All animal studies were approved by the Institutional Animal Care & Use Committee (IACUC) Program at UCSF/Gladstone.

### METHOD DETAILS

#### Hypoxia Treatment

Mice were housed in 472L acrylic boxes. 11% FiO_2_ was obtained by mixing a constant flow of nitrogen and air generated by splitting room air using a LABTEK 4CPx-P nitrogen generator. Soda lime was added to the chambers as a CO_2_ scavenger. The oxygen concentration was measured at the outlet port of the chamber with an O_2_ sensor (Vernier) which was calibrated monthly. Humidity was maintained at 30–70%. A standard light-dark cycle of ~12h light exposure was used. Mice were housed in cages with standard bedding and given unlimited access to food and water.

#### HypoxyStat Treatment

Mice were administered HypoxyStat at 600 mg/kg every other day or 400mg/kg daily via oral gavage. For the disease prevention experiments, KO mice that underwent daily dosing were dosed starting ~P30 for 1 week of 200mg/kg daily, followed by 400mg/kg daily to allow for gradual dose escalation. For the reversal study, KO mice were dosed once with 200mg/kg on postnatal day ~P55, followed by 400mg/kg daily. HypoxyStat was formulated as a suspension at 60 mg/mL, 40mg/ml or 80mg/ml depending on dose in 0.5%HPMC and stored at 4 °C. The formulation was determined to be stable for at least 8 weeks. Animal body weight was taken at time of dosing to determine appropriate dosing volume (10 mL/kg for every other day dosing, 5ml/kg for daily dosing). Vehicle treated mice were administered the same volume of 0.5% HPMC.

#### Brain Histology

Mice were euthanized via CO_2_ inhalation. To perfuse the whole body, the right atrium was nicked and a needle was inserted into the left ventricle. Ice-cold PBS was slowly injected first, followed immediately by 4% PFA solution. The brain was removed, stored in 4% PFA for 24–48 h, and then washed with PBS for 5 min (3 times). The brains were placed in 70% ethanol followed by paraffin embedding. For paraffin sections, brains were sagittally sectioned at 5 μm thickness. Prior to staining, sections were deparaffinized. Antigen retrieval was performed by heating the slides to boiling temperature in 10 mM sodium citrate solution (pH 6) in a microwave, followed by heating for 10 minutes. The sections were rinsed 3 times for 5 minutes each with PBS. They were then incubated in a blocking solution containing 5% donkey serum (DS) in PBS-Tx (PBS with 0.2% Triton X-100) for 60 minutes at room temperature. After blocking, sections were incubated overnight at 4C with the primary antibody IBA1 (Genetex GTX100042, 1:250) and NeuN (Sigma, MAB377, 1:500). Afterward, the sections were rinsed 3 times for 5 minutes with PBS-Tx. Next, they were incubated with a fluorophore-conjugated secondary antibodies (Invitrogen, A21206 or A31570) for 1 hour at room temperature. Following secondary antibody incubation, the sections were rinsed 3 times for 5 minutes with PBS-Tx, and then once for 5 minutes with PBS. Histology was quantified using either the percentage of Iba1+ positive signal or blinded scoring (on the scale of 1–5).

#### Open-Field

The open field instrument (San Diego Instruments) was used to measure spontaneous locomotor activity. Mice were placed in open field chambers and allowed to acclimate for 5 min before movement tracking for 20min. Spontaneous locomotor activity was measured based on beam breaks and recorded by the instrument. Age of measurements is +/− 5 days for practical purposes without any age bias between groups.

#### Oxy-Hemoglobin Dissociation Curve

Oxy-hemoglobin dissociation analysis was performed by the UCSF RBC laboratory using the TCS Hemox Analyzer (TCS Medical Products Company, Huntington Valley, PA). Initial pO_2_ is set to 160 mmHg. Hemoglobin is fully oxygenated under these conditions (HbO% =100). Replacement of air with nitrogen lowers pO_2_ and is measured by Clark electrode along with the change in the absorbance spectrum of hemoglobin. At a pO_2_ of 0 mmHg, hemoglobin is fully deoxygenated (HbO% =0). The relation between pO_2_ and HbO% is plotted, and the pO_2_ at which HbO% = 50% is defined as P50. The typical sigmoid de-oxygenation curve of normal blood with a P50 of 26–28 mmHg is used as a control for incubation conditions and to ensure proper function of the Hemox analyzer. For left-shifter comparison, the compounds were dissolved in HEPES buffered saline and then incubated for at least one hour at 37C with whole blood before Hemox analysis. A control of whole blood incubated with vehicle is also reported.

#### Molecular Docking

The GBT-440 structure was first prepared using Schrodinger’s structure preparation wizard where protons were added, H-bonds were optimized, and waters were removed. The structure underwent restrained minimization with OPLS4 force field. The ligand was then modified to HypoxyStat in the binding pocket followed by further restrained minimization. Published GBT-440 complex structure was used for GBT-440 image (pdb:5e83 reference: Oksenberg, D. et al. 2016 Br. J Haematol 175, 141-153).

#### PK (Half-life, blood/plasma ratio)

Mouse plasma and whole blood samples were processed as outlined below. An equal volume of 3% aqueous formic acid in water was added to whole blood and plasma samples which were then vortexed at 600 rpm for 60 minutes at room temperature. Internal standard solution (acetonitrile containing 200 nM chrysin) was then added to the samples. They were then vortexed and centrifuged for 5 minutes at 4100 rpm and 4°C. Diluted extracts were injected into a Sciex 5500 LC-MS/MS system operated in positive mode for analysis. A Waters Acquity BEH C18 (50 x 2 mm, 1.7 μM) UPLC column was used with Mobile Phases A (water:acetonitrile:formic acid [95:5:0.1, v:v:v]) and B (methanol:acetonitrile:formic acid [50:50:0.1, v:v:v]) to separate analytes. Multiple reaction monitoring transitions of 368/230 and 255/153 were used for HypoxyStat and chrysin, respectively.

#### Epo measurements

Plasma Epo measurements were performed with R&D Systems Epo Elisa Kit (MEP00B) according to manufacturer directions. Briefly, whole blood was collected with anticoagulant before centrifugation within 30 minutes of collection and stored at −80C until day of analysis. On the day of Epo assay, samples were thawed and diluted 2-fold and incubated in the assay plate for two hours. Each well was washed five times before the addition of Epo Conjugate followed by a two-hour incubation. Each well was washed five times before adding Substrate Solution to develop for 30 minutes before addition of Stop Solution. The optical density of each well was determined at 450nm and correction wavelength of 540/570.

#### Hematocrit and blood glucose, and whole blood chemistry measurements

Hematocrit was determined with an iStat VetScan 1 with Zoetis Chem 8+ cartridges. Blood glucose measurements were performed using OneTouch UltraPlus glucometer. Whole blood chemistry measurements were performed on a VetScan HM5.

#### HIF Target measurements in brain

Half of the hindbrain was collected at necropsy and immediately flash frozen. For RNA extraction, a stainless-steel bead (Qiagen) and 1 mL TRIzol^™^ Reagent (Thermo Fisher Scientific) were added to the frozen samples, which were rapidly lysed using a tissue lyser (Qiagen) for five 1-minute cycles of 30 Hz frequency. Subsequent steps for RNA isolation were identical as previously described.^9^ Reverse transcription was performed using the QuantiTect Reverse Transcription Kit (Qiagen) instructions. Briefly, gDNA was wiped out from samples containing 1 μg RNA. gDNA-free RNA samples were reverse transcribed following Qiagen’s instructions. Resulting cDNA was diluted 5-fold in DEPEC water and stored at −20°C.For each qPCR reaction, 5.5 μL Maxima SYBR Green/ROX qPCR Master Mix (Thermo Fisher Scientific) was mixed with 2.7 μL water, 0.3 μL of forward and reverse primer mix (0.3 μM in final solution), and 1.5 μL of diluted cDNA in a well of a 384-well plate. Each sample was run in triplicate. The plate was then sealed and inserted into a QuantStudio 5 (Applied Biosystems, Thermo Fisher Scientific) to determine cycle threshold (CT) values. Thermal cycle scheme was: 50°C for 2 minutes, 95°C for 10 minutes, then 40 cycles of 95°C for 15 seconds and 60°C for 1 minute, followed by 95°C for 15 seconds, 60°C for 1 minute, and 95°C for 15 seconds. For each set of triplicates, an average CT value was obtained, and the difference in average CT between the target gene and β-actin was calculated (ΔCT). Next, the difference in ΔCT value between each sample and the average of the normoxia samples was calculated (ΔΔCT). Relative expression of each gene was determined by calculating 2^−ΔΔCT^.

#### Reaction Bio InVEST Panel

##### Acetylcholinesterase Assay

This assay utilizes colorimetry to monitor the activity of acetylcholinesterase (AChE). Enzyme activity is measured by the conversion of acetylthiocholine to thiocholine, which is detected through an enhanced Ellman’s reaction. In each well, 20 μL of Assay Buffer (75.4 mM Na2HPO4, 24.6 mM NaH2PO4, pH 7.5) was added to wells designated as background/control (no enzyme). Subsequently, 20 μL of 0.2 U/mL AChE was added to the remaining wells. The reaction mixture was incubated at room temperature for 15 minutes on a shaker to allow for enzyme and drug interaction. To initiate the improved Ellman’s reaction, 30 μL of buffer containing 650 μM DTNB and 650 μM acetylthiocholine was added to each well. The color intensity, which is proportional to enzyme activity, was measured at 412 nm using an Envision 2105 plate reader.

##### COX1/2 Assay

This assay utilizes colorimetry to monitor the activity of cyclooxygenases COX-1 and COX-2, which are bifunctional enzymes exhibiting both COX and peroxidase activities. The peroxidase activity is measured colorimetrically by monitoring the appearance of oxidized TMPD at 590 nm. To begin, 170 μL of Assay Buffer (0.1 M Tris-HCl, pH 8) containing Hemin was added to wells designated as background/control (no enzyme), while 170 μL of Assay Buffer + Hemin + COX enzyme was added to the remaining wells. The plate was briefly shaken and then allowed to incubate at room temperature for 5 minutes. Following this, 20 μL of Colorimetric Substrate was added, followed quickly by 20 μL of Arachidonic Acid. After a brief shake, the plate was incubated for 2 minutes. Absorbance was then measured at 590 nm using an Envision 2105 plate reader (Revvity).

##### ERG Assay

This assay uses fluorescence polarization to monitor binding to the hERG channel. A fluorescently labeled Tracer binds to the membrane preparation containing hERG, and the displacement of the tracer occurs through competition for hERG channel binding. The membrane preparation was prepared in freshly prepared Reaction Buffer (25 mM Hepes, pH 7.5, 15 mM KCl, 1 mM MgCl2, 0.05% PF-127, and 1% DMSO). Compounds in DMSO were then delivered into the membrane mixture using Acoustic Technology (Echo 550, LabCyte Inc., Sunnyvale, CA) in the nanoliter range, followed by incubation for 10-15 minutes at room temperature. After incubation, the Tracer was added to initiate the reaction. The mixture was then incubated for 3 hours at room temperature in the dark with gentle mixing. Fluorescence polarization was measured at Ex/Em = 531 nm FP/595 nm P & S, and mP values were calculated.

##### Lck Kinase Assay

The ADP-Glo^™^ assay is a luminescent method used to monitor ADP-producing biochemical reactions. Compounds are pre-incubated with lipid kinases and substrate mixtures for 20 minutes at room temperature, followed by the initiation of the reaction with the addition of ATP. The ADP-Glo^™^ reagents then terminate the reaction in two steps: first, by depleting the remaining ATP, and second, by converting the generated ADP to ATP, which produces light through a luciferase/luciferin reaction. The luminescent signal generated is proportional to the ADP concentration and correlates with kinase activity. The kinase was prepared in freshly prepared Reaction Buffer (20 mM Hepes, pH 7.5, 10 mM MgCl2, 1 mM EGTA, 0.01% Brij35, 100 μM Na3VO4, 0.02 mg/mL BSA, 2 mM DTT, 2 mM MnCl2, 1% DMSO), with kinase without substrate being delivered to background wells. The substrate was added to the kinase solution, mixed gently, and transferred to the assay wells. Compounds in 100% DMSO were then delivered into 5 μL of the kinase reaction mixture using Acoustic Technology (Echo550, nanoliter range) and incubated for 20 minutes at room temperature, with light-sensitive precautions. ATP (0.1 μL) was then added to initiate the reaction, and the mixture was incubated for 60 minutes at room temperature. The reaction was quenched with 5 μL of ADP-Glo reagent and incubated for 40 minutes, followed by the addition of 10 μL of Detection Mixture, which was incubated for another 30 minutes. Luminescence was then measured.

##### Monoamine Oxidase (MAO) Assay

The activity of Monoamine Oxidase (MAO) was measured using a fluorescence-based assay employing the Amplex^®^ Red detection system, which detects the oxidative deamination of monoamines by MAO, resulting in the production of hydrogen peroxide (H_2_O_2_). The H_2_O_2_ reacts with Amplex^®^ Red in the presence of horseradish peroxidase (HRP) to produce the fluorescent compound resorufin. The assay was performed as follows: a 2X enzyme solution was prepared and dispensed into the wells of a reaction plate. Test compounds dissolved in 100% DMSO were delivered into the enzyme mixture using acoustic technology (Echo550; nanoliter range), followed by brief centrifugation and pre-incubation for 15 minutes at room temperature. A 2X substrate mixture was then added to all wells, except for the “No Substrate” control wells, which received only buffer, and the plate was centrifuged and shaken to ensure proper mixing. The reaction was incubated for 1 hour at room temperature. For detection, a pre-mixed solution of HRP and Amplex^®^ Red was added to the wells, and fluorescence measurements were taken kinetically using an Envision multimode plate reader over a 30-minute period, with readings at 5-minute intervals (excitation/emission wavelengths of 535/590 nm). Once the fluorescence signal reached a plateau, an endpoint reading was recorded for analysis.

##### Phosphodiesterase Assay

The assay measures AMP/GMP production resulting from enzyme activity using fluorescence polarization, which detects the displacement of a tracer bound to an AMP/GMP-specific antibody. For this assay, recombinant human PDE3A (SinoBiological, cat# P91-31G, Gene Accession# NM_001197221; amino acids 2-507-end) was expressed in *Sf9* insect cells using a baculovirus expression system and an N-terminal GST tag, with a molecular weight of 84 kDa. Recombinant human PDE4D2 (SinoBiological, cat# 60048, Gene Accession# NM_000921; amino acids 669-end) was also expressed in *Sf9* insect cells using a baculovirus expression system and an N-terminal GST tag, with a molecular weight of 84 kDa. These recombinant proteins were used in the assay to evaluate their respective enzymatic activities via AMP/GMP production.

##### Radioligand Binding Assay

The assay was performed using a reaction buffer consisting of 50 mM Tris-HCl (pH 8.0), 3 mM MgCl_2_, 0.5 mM CaCl_2_, and 0.1% bovine serum albumin. Radioligand tracer, cold reference compounds, and test compounds were prepared in the reaction buffer at 3X final concentration. Cold compounds were added to a 96-well polypropylene plate, followed by the radioligand tracer. Membrane preparations, prepared in reaction buffer, were added to initiate the reaction, which was incubated for 120 minutes on a rotating platform to achieve equilibrium. The reaction was terminated by rapid filtration under vacuum through a 0.5% PEI-treated 96-well filter plate, followed by six washes with ice-cold 50 mM Tris buffer. The filter plate was allowed to air-dry overnight, and a BackSeal was applied. To each well, 100 μL of MicroScint-0 was added, and the activity was measured on a TopCount reader 4 hours later.

## Supplementary Material

1

Supplemental information can be found online at https://doi.org/10.1016/j.cell.2025.01.029.

## Figures and Tables

**Table:** KEY RESOURCES TABLE

REAGENT or RESOURCE	SOURCE	IDENTIFIER
Antibodies		
Iba1 antibody	Genetex	RRID: AB_1240434
Anti-NeuN Antibody, clone A60	Sigma-Aldrich	RRID: AB_2298772
Donkey anti-Rabbit IgG (H+L) Highly Cross-Adsorbed Secondary Antibody, Alexa Fluor^™^ 488	Invitrogen	RRID: AB_2535792
Donkey anti-Mouse IgG (H+L) Highly Cross-Adsorbed Secondary Antibody, Alexa Fluor^™^ 555	Invitrogen	RRID: AB_2536180
Chemicals, peptides, and recombinant proteins		
HypoxyStat	WuXi Apptec	N/A
Critical commercial assays		
EPO Elisa Kit	R&D Systems	Cat#: MEP00B
OneTouch UltraPlus	OneTouch	N/A
InVEST Panel	Reaction Bio	https://www.reactionbiology.com/services/adme-safety/in-vitro-safety-screening/
VetScan HM5	VetScan	https://www.zoetisus.com/products/diagnostics/instruments/vetscan-hm5
Chem 8+	Zoetis	Cat#: 09P31-26
Experimental models: Organisms/strains		
Ndufs4 KO	Jax Labs	RRID: IMSR_JAX:027058
Oligonucleotides		
Adm Forward: ATGTCTCAGCAAGGTGTAAGG	This paper	N/A
Adm Reverse: TTCTTCATCCACAGGCGATAAT	This paper	N/A
Angpt2 Forward: ACAGCTGTGATGATAGAGATTGG	This paper	N/A
Angpt2 Reverse: CGAGTCTTGTCGTCTGGTTTAG	This paper	N/A
Depp1 Forawrd: CTCTCTCTAGCTTTCCTCTCCT	This paper	N/A
Depp1 Reverse: GCTAGACTGTCATCGCTCTTT	This paper	N/A
Acer2 Forward: GTGCGAGGACAACTACACTATC	This paper	N/A
Acer2 Reverse: CACATGCAGATGGGAGGTAAA	This paper	N/A
Vegfa Forward: TGGTTCTTCACTCCCTCAAATC	This paper	N/A
Vegfa Reverse: GGTCTCTCTCTCTCTTCCTTGA	This paper	N/A
Software and algorithms		
Prism	GraphPad	https://www.graphpad.com/
Photobeam Activity System-Open Field	San Diego Instruments	https://sandiegoinstruments.com/product/pas-open-field/
Phoenix 64	Certara	https://www.certara.com/software/phoenix-winnonlin/
QuantStudio 5	Applied Biosystems	https://www.thermofisher.com/order/catalog/product/A34322
Protein Preparation Wizard	Schrodinger	https://www.schrodinger.com/life-science/learn/white-papers/protein-preparation-wizard/

## Highlights

- Small molecules that increase oxygen-hemoglobin binding affinity cause tissue hypoxia
- HypoxyStat causes tissue hypoxia comparable to 4,500 m altitude or 11% inhaled O_2_
- HypoxyStat prevents/reverses disease in the premier mitochondrial disease mouse model
