# Supplementary material for: HypoxyStat, a small-molecule form of hypoxia therapy that increases oxygen-hemoglobin affinity
Source: Cell. Author manuscript; Available in PMC 2025 Jun 24. (PMC12186697; doi:10.1016/j.cell.2025.01.029)
Supplement: 1 [file NIHMS2087898-supplement-1.pdf]

CellPress
OPEN ACCESS

Supplemental figures

Dose[a]	[HypoxyStat][b]	[Hb][c]	% Hb occupancy[d]
200 mpk	0.79 mM	17.3 g/dL (2.69 mM)	29.3
600 mpk	1.19 mM	18.5 g/dL (2.87 mM)	41.4

Figure S1. Hemoglobin occupancy estimates, related to Figure 1

LC/MS/MS measurement of steady-state whole-blood HypoxyStat concentrations after day 12 administration in C57BL/6 mice (200 or 600 mg/kg PO, 3× per week in 0.5% HPMC), *N* = 10 per group.

(A) Oral administration of HypoxyStat every other day (vehicle = 0.5% HPMC).

(B) LC/MS/MS measurement of steady-state mean whole-blood HypoxyStat concentrations after day 12 administration in C57BL/6 mice.

(C) Hb concentration after analogous treatment.

(D) Hb occupancy was calculated by dividing the HypoxyStat concentration in blood by the Hb concentration in blood on day 12.

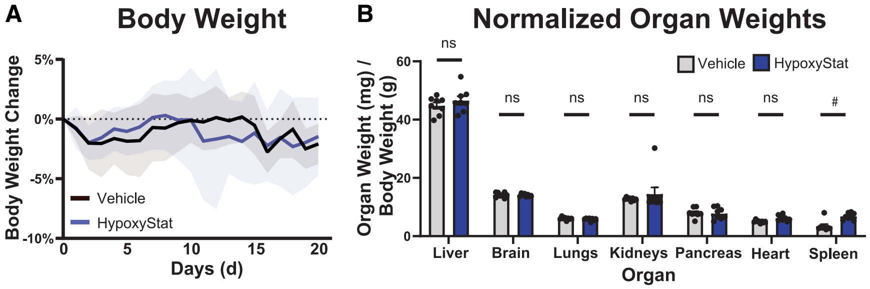

C **Clinical Chemistries and Complete Blood Count**

Test	Vehicle Mean	Vehicle StDev	HypoxyStat Mean	HypoxyStat StDev	Significance
Na mmol/L	150.0	22.3	132.1	2.0	ns
Cl mmol/L	113.7	1.3	114.1	2.5	ns
TCO2 mmol/l	23.6	1.4	20.5	1.5	ns
BUN mg/dl	30.0	5.1	61.6	8.0	ns
WBC 10^9/L	10.0	3.3	11.2	1.6	ns
LYM 10^9/L	8.8	3.0	9.4	1.1	ns
MON 10^9/L	0.3	0.3	0.4	0.3	ns
NEU 10^9/L	1.0	0.5	1.4	0.7	ns
MCV fl	40.6	0.5	50.4	1.3	ns
MCH pg	13.2	0.2	16.6	0.6	ns
MCHC g/dl	32.5	0.7	33.0	0.6	ns
RDWc %	20.4	0.9	24.2	0.6	ns
RDWs %	30.3	1.3	44.4	1.3	ns
PLT 10^9/L	461.8	131.8	265.5	79.3	#
MPC fl	5.9	0.1	6.8	1.5	ns
PCT %	0.3	0.1	0.2	0.1	ns
PDWc %	29.8	1.5	34.2	3.0	ns
PDWs fl	7.1	0.7	10.1	2.6	ns

significant and known to be affected by hypoxia

Figure S2. No overt toxicity observed by HypoxyStat (body weight, organ weights, clinical chemistries, and CBC), related to Figure 3
(A–C) (A) Percent change in body weight (mean ± SD), (B) organ weights normalized to body weight (ANOVA), and (C) clinical chemistries and complete blood count (CBC) for mice treated daily with vehicle or HypoxyStat for 3 weeks. Student t test. # Indicates a significant and known adaptation with inhaled hypoxia.

A

HypoxyStat does not Inhibit Common Off-Target Proteins

		% Activity		IC50 (M)	Reference	Signficant inhibition (50% or greater)
		HS	HS	Reference	Compound	
Target Family	Target	Data 1	Data 2	Compound		
G-protein Coupled Receptors (GPCRs)	5-HT1A	75	65	1.01E-08	8-OH DPAT	Negative
	5-HT1B	114	117	1.73E-08	GR125743	Negative
	A2A adenosine	126	161	1.72E-08	CGS21680	Negative
	α1A adrenergic	88	111	9.93E-09	Prazosin	Negative
	α2A adrenergic	78	110	1.66E-08	RX 821002	Negative
	β1 adrenergic	102	108	4.07E-08	Alprenolol	Negative
	β2 adrenergic	89	111	1.01E-07	Alprenolol	Negative
	CCK	104	101	8.12E-08	Lorglumide	Negative
	H1 histamine	87	88	7.88E-09	Mepyramine	Negative
	Muscarinic M1	108	117	1.53E-08	Pirenzepine	Negative
	Muscarinic M2	77	90	3.51E-08	AF-DX 384	Negative
	Muscarinic M3	87	80	2.63E-08	4-DAMP	Negative
	δ opioid	97	101	5.25E-09	DADLE	Negative
	κ opioid	140	126	1.05E-08	U-69,593	Negative
	μ opioid	73	85	1.28E-08	DAMGO	Negative
Transporters	DAT	91	87	1.07E-07	AHN 1-055	Negative
	SERT	112	74	7.36E-08	Imipramine	Negative
Ion Channels	5HT3	51	65	7.08E-09	Quipazine	Negative
	Central BZD	100	104	1.12E-08	Flumazenil	Negative
	hERG	69	71	1.61E-08	E-4031	Negative
Kinase	Lck	82	85	3.45E-09	Captopril	Negative
Phosphodiesterase	PDE3A	93	93	1.40E-05	IBMX	Negative
	PDE4D2	88	90	1.42E-05	IBMX	Negative
Cyclooxygenase	COX-1	91	88	1.06E-08	SC-560	Negative
	COX-2	86	86	6.96E-08	DuP-697	Negative
Cholinesterase	Acetylcholinesterase	105	106	5.98E-08	Physostigmine	Negative
Monoamine Oxidase	MAO-A	102	102	1.52E-07	Tranylcypromine	Negative
Nuclear Receptor	Glucocorticoid Receptor	91	95	2.55E-09	Dexamethasone	Negative

Figure S3. Reaction Bio InVEST panel for off-targets does not detect any adverse binding of HypoxyStat, related to Figure 3
(A) The category of off-targets, name of off-targets, percent activity, reference compounds, and significance of off-target binding by HypoxyStat. All assays completed in duplicate. Significance defined by 50% or greater inhibition in both replicates (standard industry criteria used for this assay).

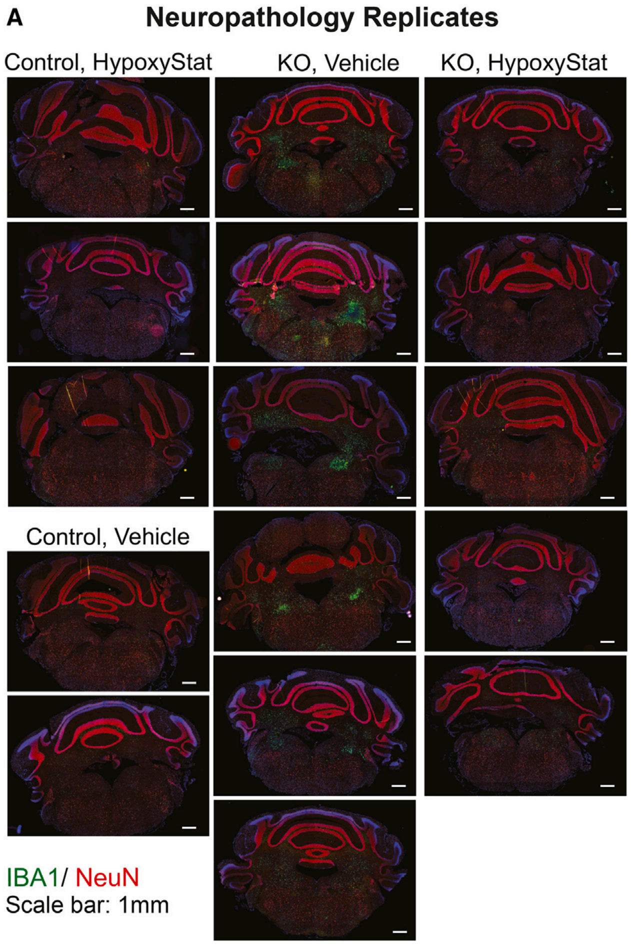

Figure S4. Neuropathology for vehicle and HypoxyStat-treated KO and WT mice (replicates), related to Figure 4
Iba1 staining (green) of WT and KO mice treated with HypoxyStat or vehicle starting P30. Brains harvested at ~P50–P55 in all groups. DAPI co-staining in blue and NeuN in red. Images shown for multiple animals per group. Replicates shown, including samples included in Figure 4.

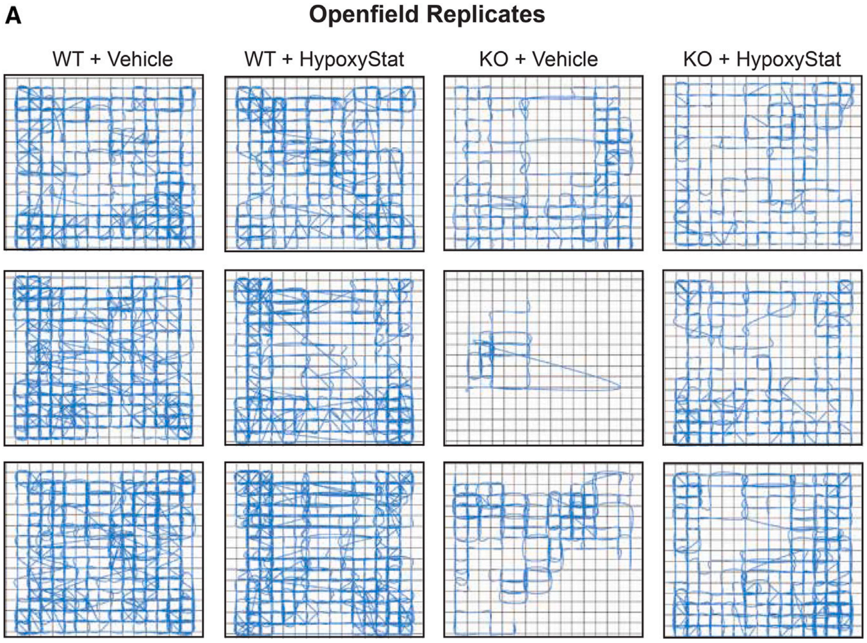

Figure S5. Open-field traces for vehicle and HypoxyStat-treated KO and WT mice (replicates), related to Figure 4
Spontaneous movement of WT and KO mice treated with HypoxyStat or vehicle starting at P30. Experiment performed at ∼P50–P55 in all groups. Images shown for representative triplicate animals per group.

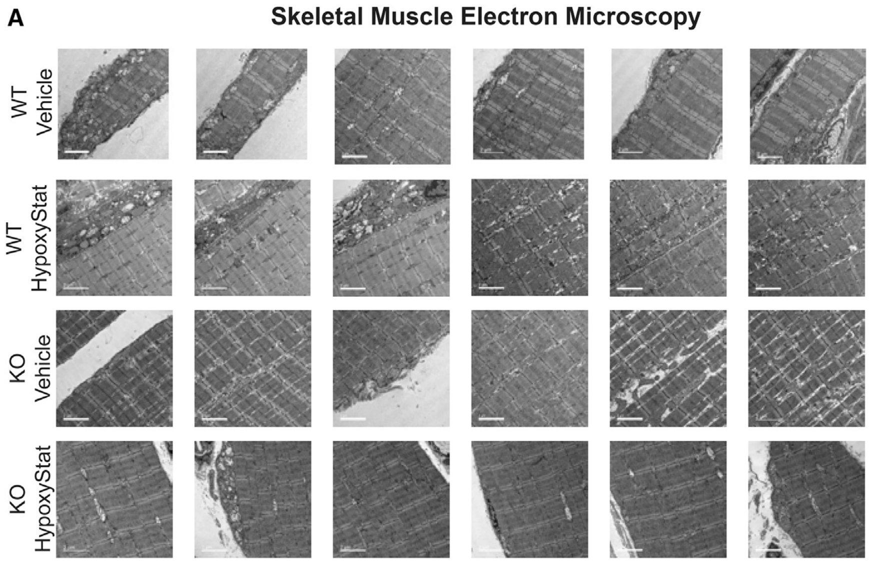

Figure S6. Skeletal muscle electron microscopy in *Ndufs4* KO and WT mice +/− HypoxyStat, related to Figure 4

(A) Six representative images are provided of EM of skeletal muscle in KO vs. WT mice treated with vehicle or daily HypoxyStat for ~25 days.
